# Diet, physical exercise and cognitive behavioral training as a combined workplace based intervention to reduce body weight and increase physical capacity in health care workers - a randomized controlled trial

**DOI:** 10.1186/1471-2458-11-671

**Published:** 2011-08-27

**Authors:** Jeanette R Christensen, Anne Faber, Dorte Ekner, Kristian Overgaard, Andreas Holtermann, Karen Søgaard

**Affiliations:** 1 Department of Sport Science, Aarhus University, Aarhus, Denmark; 2 National Research Centre for the Working Environment, Copenhagen, Denmark; 3 Institute of Sports Science and Clinical Biomechanics, University of Southern Denmark, Odense, Denmark

## Abstract

**Background:**

Health care workers comprise a high-risk workgroup with respect to deterioration and early retirement. There is high prevalence of obesity and many of the workers are overweight. Together, these factors play a significant role in the health-related problems within this sector. The present study evaluates the effects of the first 3-months of a cluster randomized controlled lifestyle intervention among health care workers. The intervention addresses body weight, general health variables, physical capacity and musculoskeletal pain.

**Methods:**

98 female, overweight health care workers were cluster-randomized to an intervention group or a reference group. The intervention consisted of an individually dietary plan with an energy deficit of 1200 kcal/day (15 min/hour), strengthening exercises (15 min/hour) and cognitive behavioral training (30 min/hour) during working hours 1 hour/week. Leisure time aerobic fitness was planned for 2 hour/week. The reference group was offered monthly oral presentations. Body weight, BMI, body fat percentage (bioimpedance), waist circumference, blood pressure, musculoskeletal pain, maximal oxygen uptake (maximal bicycle test), and isometric maximal muscle strength of 3 body regions were measured before and after the intervention period.

**Results:**

In an intention-to-treat analysis from pre to post tests, the intervention group significantly reduced body weight with 3.6 kg (p < 0.001), BMI from 30.5 to 29.2 (p < 0.001), body fat percentage from 40.9 to 39.3 (p < 0.001), waist circumference from 99.7 to 95.5 cm (p < 0.001) and blood pressure from 134/85 to 127/80 mmHg (p < 0.001), with significant difference between the intervention and control group (p < 0.001) on all measures. No effect of intervention was found in musculoskeletal pain, maximal oxygen uptake and muscle strength, but on aerobic fitness.

**Conclusion:**

The significantly reduced body weight, body fat, waist circumference and blood pressure as well as increased aerobic fitness in the intervention group show the great potential of workplace health promotion among this high-risk workgroup. Long-term effects of the intervention remain to be investigated.

**Trial registration:**

ClinicalTrials.gov: NCT01015716

## Background

Overweight and obesity are well documented to be associated with major chronic illnesses, including hypertension, diabetes, arthritis, heart diseases, cancer and all-cause mortality [1-3]. Moreover, excessive body weight has also been shown to increase the risk for musculoskeletal pain [4], sick leave [5] and early retirement from the workforce before they are entitled to state pension [6], causing high socioeconomic costs [7]. Effective interventions for weight reduction and addressing obesity are therefore a high priority.

It is well documented that being overweight or obese is inversely associated with educational level and occupational class in developed countries [8], particularly among women [9]. Because education and gender often works as stratification into certain labor market sectors, workplaces may be optimal arenas for reaching high-risk populations for overweight and obesity. Health care workers represent a high risk population with high physical demands, involving patient handling and other manual work tasks with high peak force, walking and standing as well as awkward postures [10]. Health care work is predominantly performed by female employees with high prevalence of overweight and low physical capacities and a high prevalence of musculoskeletal pain [11]. Studies suggests, it may be the combination of high body weight, low physical capacity and high physical work demands that causes the high prevalence of musculoskeletal pain [12-15]. Effective well-documented initiatives for reducing weight, improving physical capacity and reducing musculoskeletal pain among health care workers are therefore needed.

Strength training has been shown to improve physical capacity and reduce musculoskeletal pain [16]. Meanwhile, different strategies to reduce overweight have been suggested, as well as several consensus statements regarding weight loss maintenance for individualized interventions, for taxes, tariffs and trade laws policies, and the built environment [17,18]. Diet alone has shown limited effectiveness for long term weight loss maintenance [19]. Programs combining diet and physical exercise are therefore recommended to avoid reductions in energy metabolism with dietary restrictions [20]. Grave and colleagues suggest that weight regain is due to failure to keep up physical activity, as maintenance of physical activity is fundamental for long-term weight loss [21]. The key to maintaining physical activity is new cognitive procedures and strategies that will help weight-loser's to build a mind-set of long-term weight control. In summary, more multidisciplinary interventions are recommended [21] and should include a combination of the three elements - dietary change, physical exercise and cognitive behavioral training [22]. However, only few studies have combined these initiatives [23] and to our knowledge, no previous studies have investigated the combined effects of these initiatives on weight loss at a high-risk group like health care workers in a workplace setting.

Therefore, the main aim of this study was to investigate the effects of a workplace intervention combining diet, physical exercise and cognitive behavioral training on body weight, general health variables and physical capacity in health care workers. The secondary aim was to study if these health promotions could affect musculoskeletal pain among health care workers. This paper presents results from the first three months of a one year intervention.

## Methods

### Study design

The study is part of the FINALE program, which has the long-term aim to reduce physical deterioration indicated by musculoskeletal disorders, work ability and sickness absence among workers with high physical work demands. Details of the background, design and conceptual model of FINALE are previously reported [24]. The present study, FINALE-health is a cluster randomized single-blinded controlled trial conducted from May 2009 to the end of June 2010. The 14 months included 12 months intervention with tests performed at baseline, after three months, and after one year. In this paper, the effects of the first three months of intervention are reported. All participants worked as personnel in care units in the western part of Denmark. The project was ethically approved by The Central Denmark Region Committees on Biomedical Research Ethics (M-20090050), and qualified for registration in the International Standard Randomized Controlled Trial Number Registry (NCT01015716).

### Workplace recruitment

Initially, three Danish municipalities in Central Jutland (DK) were contacted. Randers municipality agreed to participate immediately, and the project was initiated there. Randers Municipality consisted of nine care areas that were considered for the project. For practical implementation of the intervention, only care areas meeting inclusion criteria of at least 50 health care workers with a minimum employment of 15 hours/week were considered. Furthermore, it was requested that the care area was not involved in other health related projects. Four care areas had too few health care workers, three were already involved in health projects, and one suddenly acquired new management and could not find resources to enroll in the project. This left one care area that was eligible to participate.

### Recruitment procedure and study population

It was a management demand that all employees working at least 15 hours/week should be invited to participate in the project. The recruitment of participants was therefore based on the complete payroll of employees. Three introductory one-hour meetings were held in April and May 2009, and a total of 159 (out of 202 invited) employees attended one of these meetings. The employees filled out a screening questionnaire handed out at the meetings, with questions about health, and if they wanted to participate in the study. Employees, who did not attend the meetings, were given written information and screening questionnaires from their closest manager. Questionnaires and possible consent were returned in a sealed envelope. The primary target group for the intervention included those fulfilling the following inclusion criteria; being female and overweight, defined as BMI > 25 or having body fat % > 33 (age 18-40 years) or > 34 (age > 40 years) [25] as well as working primarily as health care workers, or with elderly care as primary work task.

**Figure 1:**
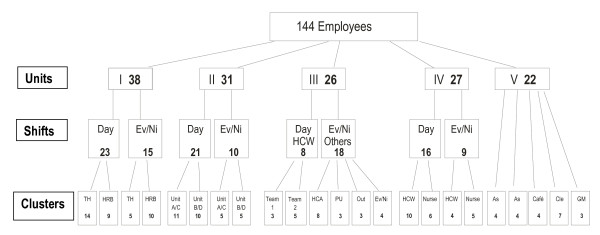
**Flow of the cluster randomization**. Cluster flow of consenting employees stratified in levels of work units (Units), day and evening/night shifts (Shifts) and clusters for randomization based on daily contact at work between participants (Clusters). Day = Day shifts, Ev/Ni = Evening/Night shifts, HCW = Home Care Worker, AS = Administration Staff, Cle = Cleaners, GM = Grounds Men, TH = Home Care Workers in a Terraced House, HRB = Home, Care Workers in a high-rise block, HCA = Health Care Assistants, PU = Psychiatric Unit.

All who consented to participate in the study were subsequently invited for a physical test the following week and through their closest manager handed a questionnaire, to be returned at the test session. Help with understanding the questionnaire was offered by the test leader of the physical tests, which took place during working hours at the worksite. Subsequently, the consenters were enrolled and randomly allocated to either intervention or reference group. For an overview of the procedure, see Figure 1.

### Cluster-randomization procedure

Groups were created based on information from the screening questionnaire and the management of working teams, day and evening/night shifts and close working relations. This approach was chosen to avoid contamination between the intervention and the reference group, and so that the participants could benefit from the social support of colleagues in their unit. The aim was to increase compliance and to facilitate the necessary practical arrangement at the work place. It was therefore decided to integrate the intervention into work time. A cluster formation of the groups was performed to assure equal allocation in the intervention and reference groups balanced on sex, age, job seniority or job type with cluster size varying from 3 to 15. The randomization was done by an external research group, which had no knowledge of the work place or the participants. Clusters were randomly allocated to intervention and control by the drawing of sealed envelopes from a bag. An overview of the resulting allocation is given in Figure 1.

### Intervention

The intervention lasted 12 months and consisted of two parts. The first part *(0-3 months) *focused on weight loss and included advice on dietary change based on the Danish Dietary recommendations, calorie counting, weight measurements, weight loss targets, strengthening exercises and initiating leisure time fitness exercise. The remaining part that focused on weight loss maintenance *(3-12 months) *is not described in this paper.

### Instructors and Instruction

Instruction was given as a weekly one hour session during work time. The intervention group consisted of 70 participants divided into seven training teams - each with its own instructor. The aim was to create a close-knit team spirit, which hopefully would help prevent dropouts. The project manager (JRC) and two employed instructors with sports degrees taught the seven teams. The instructors served as substitutes for each other during holidays and sick leave. Prior to the start the instructors were acquainted with the project, its aim, hypotheses, etc. They were also encouraged to read a literature list consisting of the Danish Dietary recommendations, "Overvægtens psykologi" (The Psychology of Obesity), by Tove Hvid [26], "At tale om forandring" (Talk About Change) by the The Danish Council on Smoking and Health [27] as well as a micro-compendium describing the cognitive behavioral training specifically tailored for this intervention. The three instructors also met for a whole day before each phase, going through the phase, the materials and outlining the content of each session. At the start of the intervention, JRC supervised all training for the first month, and subsequently offered support when required. Two-hour weekly meetings were held with instructors and JRC with fixed agendas, including follow-up on the previous week's session, next week's session, team compliance and good and bad experiences.

### Dietary Intervention

A subsample of the study population filled out dietary records which were used to obtain information on dietary preferences. This information was adjusted according to the Danish Dietary recommendations, and used to create different exemplary courses with specific calorie amounts. These courses were proposed for every mealtime in amounts adjusted to suit an individual calorie prescription. To obtain an estimate of daily energy requirement, each individual's resting metabolism was calculated, based on gender, age and weight and multiplied by a Physical Activity Level factor (PAL) of 1.8 [28]. Then 1200 calories were subtracted from the estimated daily energy requirements giving an individual calorie prescription. These values were chosen to achieve a weight reduction rate of 1 kg per week [29]. If weight loss after two weeks was less than expected, the participants were given meal plans which further lowered their planned daily calorie intake by 300 kcal a day. Prescribed calorie amount was lowered in steps of 300 kcal a day throughout the intervention as participants decreased their weight. The dietary advises and the weight check occupied approximately 30 min of the weekly session.

### Physical exercise training

10 - 15 minutes physical exercise training was included in the weekly session at the workplace. Focus during sessions was on strength training to increase muscle mass in the lower extremities in order to increase resting metabolism and maintain physical capacity. These exercises consisted of both one and two legged squats, with and without dumb bells and core balls, and lunges walking forward and to each side. Other exercises focused more on general strength, and included exercises for abdominal and back extension, shoulders and arms. Participants brought home a strength training program, picturing these exercises, and were encouraged to perform them twice a week at home. In addition to the brief training sessions, participants were encouraged to initiate aerobic leisure time exercises such as biking, walking, running, swimming or attending different sports in the local area for two hours weekly. The dose of the instructed physical exercises in the sessions progressed in intensity throughout the weeks of the intervention, by increasing weights and repetitions. To motivate participants and individualize feedback from the instructors log books to monitor leisure time exercises was given to the participants and were shown to the instructor at each session.

### Cognitive behavioral training

From a cognitive behavior program, designed by Linton aiming to prevent chronic musculoskeletal pain [30], a specific cognitive behavioral training (CBT) tool were modified and tailored to support a change to a more physically active lifestyle and by addressing the distress and challenges involved with weight loss. Whereas general counseling are not obliged to follow specific methods, traditionally cognitive behavior therapy aims at reflecting on dysfunctional attitudes and coping behaviors, discussing functional alternatives, and training the implementation of these in everyday life [30]. This included helping the participants to make realistic weight loss targets, find personal strategies to ease hunger, continue healthy behaviors, cope with social contexts and situations involving alcohol, food etc. These elements were discussed in the groups based on a specifically tailored guideline, containing 15 exercises such as pro-and-con schemes and positive thinking strategies with homework between each session. The CBT was offered as a 15 min part of the weekly sessions.

#### Reference group

The reference group was offered a monthly two-hour oral lecture during working hours. The three presentations were based on the Danish National Board of Health and the Ministry of Food, Agriculture and Fisheries public websites and concerned the Danish Dietary recommendations.

## Data collection and study materials

### Objective measures

All participants were tested at baseline and after three months. Each test session lasted an hour and consisted of anthropometrical, health-related and physical capacity measures specified as the following. *Height *was measured to the nearest mm without shoes. *Body weight *was measured wearing light clothes, but without socks and shoes. One kilogram was subtracted from the weight measure to compensate for clothing. *Body Fat *was measured using a bio impedance device (TANITA SC-330), which was set to 'standard' while body frame and the participant's age, height and gender were entered. *Waist circumference *was measured over the umbilicus standing up and with clothes on, using an ergonomic circumference measuring tape (Seco 203 Girth measuring tape) and clothes thickness was noted. *Blood pressure *was measured in seated position after 10 minutes of rest with an electronic blood pressure monitoring device (Artsana CS 410). Three measurements were done one minute apart and an average calculated [31]. Aerobic fitness was measured using a Monark E327 bicycle ergometer and a pulse oxiometer (Nellcor OxiMax N-65) fitted on the ring finger. Participants cycled for five minutes at 70 watts (60 rpm, 1 kp). During the first five minutes, the test subject was asked to answer the question: "*How would you rate your fitness? Respond with one of the following classes:" *Extremely good/very good/average/poor/very poor. Hereafter load was increased by 35 watts (1/2 kp) every other minute until the test subject was forced to stop because of exhaustion. With 30 seconds to the next work-load increase, participants were asked to assess the level of perceived exertion using the Borg Scale (rate of perceived exertion on a scale from 6-20) and heart rate was measured. The total number of seconds elapsed and the subject's maximum heart rate were noted. An algorithm was used to estimate maximal oxygen uptake (VO_2_-max) [32]. VO_2_-max values were expressed either as absolute values in L O_2_/min or relative to body weight in ml O_2_/kg/min (aerobic fitness).

Isometric maximal voluntary strength was obtained with a reproducible standardized setup [33], measuring maximal voluntary handgrip, shoulder elevation, and back flexion and extension force [34]. The participants performed a minimum of three attempts with steady increasing force to reach maximum within 3-5 seconds. The test was repeated until a maximal of five contractions if the last attempt showed a more than 5% increase. The participant rested at least 30 seconds between each attempt. The maximal attempt was recorded for further analysis. Standardized verbal command and encouragement was given to maximize the effort. *Handgrip *in both hands was measured using a grip strength measurer (La Fayette) [35]. *Shoulder elevation *was measured with a Bofors dynamometer with the subject seated erect in a chair with legs hanging freely, arms hanging along the side and head facing forward. The distance from pressure point to sternoclavicular joint was measured as the moment arm [36]. Back *flexion and extension *were measured with the subject standing, facing/backing onto beam and support plate at the spina iliaca anterior superior. The Bofors dynamometer was fixed to pull horizontal with a belt positioned at the vertical level of m. deltoid insertion on the humerus. The distance from the belt to a line through the crista iliaca and lumbalcolumna (L4L5 level) was measured for the moment calculation [37].

Prior to the test session, participants were screened in accordance with the exclusion criteria for the test. The exclusion criteria for one or more of the tests were elevated blood pressure, defined as systolic values higher than 110 mmHg + age in years, or diastolic values higher than 100 mmHg regardless of age [31], angina pectoris, heart or lung prescription medication, current or pervious illnesses and trauma, herniated disc, tennis elbow, golf elbow, Carpal Tunnel Syndrome, significant level of musculoskeletal pain at the time of the test and pregnancy. The test manager was blinded regarding the participants' intervention status, and whenever possible the same test manager tested the subject both before and after the three-month intervention.

### Questionnaire

A questionnaire was completed twice, approximately one week before each test round. The questionnaire was developed for use in all workplaces participating in the FINALE program and consisted of 140 questions mainly of standardized and validated scales [24]. In the present paper, responses to questions on musculoskeletal disorders are reported. *Musculoskeletal disorders *were measured with the Nordic questionnaires of musculoskeletal disorders [38], supplemented with questions about localized pain intensity [39].

### Statistical analyses

A power calculation was carried out for the main outcomes - weight change, comparing two groups of equal size. Power was set to 0.8 with a significant level of 0.05. At least 30 participants in each group were needed to detect a difference in weight loss of at least 3 kg. With an estimated 30% drop out, 43 participants were needed in each group. PASW statistics 18 was used for the statistical analysis. Differences between intervention and reference group at baseline were tested with Pearson's x^2 ^for distribution in sex, education (health care workers), current smoking status and the dichotomized parameter for musculoskeletal symptoms in neck, shoulders, upper- and lower back. All other parameters were tested with a Student's t-test. When comparing intervention group and reference group over time, ANCOVA analysis were performed in accordance to the intention-to-treat principle, i.e. all randomized participants are included in the analyses with missing values substituted with carried forward or backwards measured variables. Clusters, age and the investigated value at baseline were included as covariates. All results are given as mean (SD). p < 0.05 are considered statistically significant.

## Results

### Employee flow

**Figure 2:**
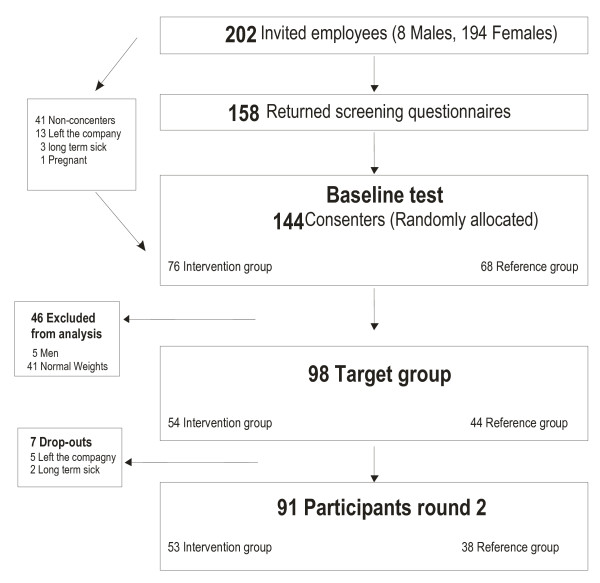
**Flow of the participants**.

A flow-chart of the project is presented in Figure 2. From the employee list, 202 persons (8 men and 194 women), working at least 15 hours/week were invited to participate in the study. Among these, 144 fulfilled the inclusion criteria and consented to participate, and were randomly allocated to either the intervention or the reference group. Among these, 139 were women, 105 worked with health care as main task, and 98 met the full criteria to enter target group (i.e. women, overweight based on BMI or fat percentage, health care workers or having similar education with daily patient care). Among the 98 female overweight health care workers, 91 were still taking part in the study after three months (five left the company and two were on long-term sick leave).

### Baseline characteristics of workplace population

The participants in the study were on average 45.5 (9.5) years of age, 77.4 (16.8) kg body weight, 36.8 (8.2)% in fat percentage, 28.1 (5.8) in BMI and 94.6 (15.0) cm in waist circumference. A BMI ≥ 25 was found for 64.5% of the employees, and a critically high waist circumference (> 88 cm) was recorded for 61.1%. Average blood pressure was 130/82 mmHg, and 31.6% had elevated blood pressure (> 139/89 mmHg) [31]. There were no anthropometrical differences between the intervention and the reference group. In Table 1, details are given on intervention and reference group within the workplace population.

**Table 1:** Anthropometric, lifestyle and work related characteristics at baseline of the whole and the target population.

		Whole population (n = 144)			Target population (n = 98)		
		**Intervention** **group** **(n = 76)**	**Reference** **group** **(n = 68)**	**P-** **values**	**Intervention** **group** **(n = 54)**	**Reference group (n = 44)**	**P-** **values**

Sex (females)	**N** %	**75** 98.7	**64** 94.1	**0.189**	**54** 100	**44** 100	**1.000**
Education (health (care worker)	**N** %	**58** 80.6	**49** 74.2	**0.302**	**41** 77.4	**35** 79.5	**0.497**
Current smoking	**N** %	**21** 32.8	**18** 32.7	**0.430**	**18** 35.3	**9** 26.5	**0.244**
Age (years)	**Mean** SD	**44,8** 9.5	**46.4** 9.5	**0.314**	**45.7** 8.7	**46.0** 8.6	**0.893**
Job Seniority (months)	**Mean** SD	**176.0** 105.2	**158.7** 122.4	**0.418**	**188.8** 105.1	**152.0** 120.9	**0.146**
Working hours (hours/week)	**Mean** SD	**32.3** 3.5	**32.0** 4.1	**0.708**	**32.1** 3.4	**32.3** 3.4	**0.744**
Height (cm)	**Mean** SD	**166.0** 5.9	**165.5** 6.9	**0.649**	**166.2** 5.8	**165.1** 6.7	**0.410**
Weight (kg)	**Mean** SD	**78.3** 17.3	**76.3** 16.3	**0.499**	**84.3** 16.0	**83.0** 14.4	**0.660**
Body mass index (kg/m^2^)	**Mean** SD	**28.4** 6.0	**27,8** 5.6	**0.558**	**30.5** 5.4	**30.4** 4.9	**0.898**
Fat percentage (%)	**Mean** SD	**37.5** 7.9	**36.0** 8.6	**0.274**	**40.9** 5.8	**40.5** 5.7	**0.744**
Waist circum-ference (cm)	**Mean** SD	**94.8** 15.0	**94.3** 15.2	**0.856**	**99.7** 13.7	**101.6** 12.4	**0.492**
Systolic blood pressure (mmHg)	**Mean** SD	**132.1** 18.6	**127.0** 13.4	**0.077**	**134.1** 19.3	**129.3** 11.9	**0.162**
Diastolic blood pressure (mmHg)	**Mean** SD	**83.7** 10.7	**80.1** 9.1	0.042*	**85.1** 10.8	**81.7** 8.3	**0.108**

Because of some missing data, the number of responders on the different measures varied from 53-76 of the whole population and 34-54 of the target group.

Where N is displayed, percentages are based on the number of responses.

### Baseline characteristics in target population

At baseline there were no significant differences between the intervention and the reference group in anthropometric measures in the target group (Table 1). In addition, physical capacity measured as muscle strength and maximal oxygen uptake was similar in the intervention and the reference groups (Table 2). Mean hand grip for both groups was 303 (54) N, shoulder elevation was 66 (24) Nm and 58 (24) Nm for right and left shoulder, respectively and trunk flexion and extension were 127 (39) Nm and 122 (40) Nm, respectively. VO_2 _max was 2.1 (0.4) l/min, and aerobic fitness was 26.2 (4.8) ml/min/kg. Corresponding data are given for intervention and reference group separately (Table 2). Musculoskeletal pain at baseline showed no difference between the intervention and the reference group in any body region. The 12 months prevalence in both groups was 75-80% for the neck and low back, while for the upper back and shoulders it was around 55%. The seven days prevalence for the neck and lower back was about 45%, with mean pain intensity of 2.5 on a scale from 0-10. The 7 days prevalence for the lower back was about 20%, with mean intensity about 2.

**Table 2:** Physical capacity and musculoskeletal pain at different body regions at baseline of the target population.

		Intervention group (n = 54)	Reference group (n = 44)	P- values
Handgrip Dom side (N)	Mean (SD)	298.9 (52.9)	308.2 (56.0)	0.420
Right shoul. elevation (Nm)	Mean (SD)	73.6 (22.9)	61.4 (24.2)	0.085
Left shoul. elevation (Nm)	Mean (SD)	62.0 (22.1)	54.7 (24.4)	0.283
Back flexion (Nm)	Mean (SD)	127.2 (31.7)	133.1 (49.9)	0.613
Back extension (Nm)	Mean (SD)	117.3 (39.9)	118.4 (42.5)	0.927
VO_2 _Max (L/min)	Mean (SD)	2.07 (0.36)	2.13 (0.34)	0.517
Physical fitness (ml/min/kg)	Mean (SD)	25.80 (4.62)	26.72 (5.12)	0.474
**Neck pain**				
Last 12 months (N = yes)	N (%)	42 (82.4)	25 (73.5)	0.282
Last 7 days (N = yes)	N (%)	23 (45.1)	12 (35.3)	0.500
Intensity last 7 days (1-10)	Mean (SD)	2.6 (2.6)	2.1 (2.4)	0.493
**Shoulder pain (right)**				
Last 12 months (N = yes)	N (%)	29 (56.9)	18 (52.9)	0.699
Last 7 days (N = yes)	N (%)	18 (36.0)	8 (23.5)	0.242
Intensity last 7 days (1-10)	Mean (SD)	2.1 (2.7)	1.0 (1.6)	0.062
**Upper back pain**				
Last 12 months (n = yes)	N (%)	30 (58.2)	18 (54.5)	0.372
Last 7 days (n = yes)	N (%)	15 (29.4)	9 (26.5)	0.811
Intensity last 7 days (1-10)	Mean (SD)	1.9 (2.3)	1.5 (2.5)	0.562
**Lower back pain**				
Last 12 months (N = yes)	N (%)	41 (80.4)	27 (79.4)	0.139
Last 7 days (N = yes)	N (%)	24 (47.1)	15 (44.1)	0.827
Intensity last 7 days (1-10)	Mean (SD)	2.6 (2.4)	2.9 (3.1)	0.651

Due to missing data, the number of responders on the different measures varies from 22-54.

Where N is displayed, percentages are based on the number of responses.

### Changes after 3 months in target population

Table 3 presents average changes in the target group from baseline to 3 months of all measures in the intervention and the reference group. A highly significant Intervention group* Test round interaction was found for weight loss, BMI, fat percentage, waist circumference and diastolic blood pressure. In the intervention group, body weight decreased from 84.2 to 80.6 kg, corresponding to a decrease in BMI from 30.5 to 29.1. Fat percentage fell from 40.9 to 39.3% and waist circumference decreased from 99.3 to 95.1 cm. Blood pressure was lowered from 134.1/85.2 to 126.6/79.8 mmHg. For the reference group, no significant changes were found except for an increased BMI from 30.4 to 30.7. Regarding physical capacity, no significant Intervention group* Test round interaction was found for muscle strength. VO_2 _max was unchanged in both groups, while an Intervention group* Test round interaction (p < 0.011) was found for aerobic fitness with the intervention group increasing from 25.9 to 28.0 ml/min/kg. Regarding musculoskeletal pain, no significant Intervention group* Test round interactions were found.

**Table 3:** Mean change from baseline to 3 months test in anthropometric characteristics of the target population.

	Intervention group (n = 54)		Reference group (n = 44)		Time vs. group interaction
	**Δ mean**	**P**	**Δ mean**	**P**	**P**

Weight (kg)	-3.59 (3.80)	0.000	+0.68 (2.37)	0.064	0.000
Body mass index (kg/m^2^)	-1.31 (1.39)	0.000	+0.27 (0.85)	0.039	0.000
Fat percentage (%)	-1.56 (2.78)	0.000	+0.33 (1.25)	0.093	0.000
Waist circumference (cm)	-4.24 (6.10)	0.000	-0.91 (4.18)	0.165	0.001
Systolic blood pressure (mmHg)	-7.52 (12.97)	0.000	-2.11 (9.25)	0.148	0.067
Diastolic blood pressure (mmHg)	-5.43 (7.79)	0.000	-0.68 (7.17)	0.543	0.016
Hand grip Dom side (N)	+10.28 (31.80)	0.022	-6.22 (33.97)	0.248	0.102
Right shoul. Elevation (Nm)	-0.03 (23.78)	0.994	+5.57 (19.41)	0.164	0.087
Left shoul. Elevation (Nm)	+1.49 (15.73)	0.548	+5.66 (18.53)	0.140	0.218
Back flexion (Nm)	+9.02 (23.94)	0.016	-3.99 (33.95)	0.555	0.068
Back extension (Nm)	+1.26 (31.56)	0.796	+18.04 (34.46)	0.013	0.045
VO_2 _(L/min)	+0.14 (0.22)	0.003	+0.01 (0.21)	0.834	0.185
Physical fitness (ml/min/kg)	+3.33 (3.48)	0.000	-0.11 (2.87)	0.889	0.011
**Intensity of musculoskeletal pain last 7 days**					
Neck	-0.67 (2.15)	0.028	-0.24 (2.24)	0.538	0.452
Shoulder (right)	-0.00 (1.59)	1.000	-0.03 (1.54)	0.913	0.427
Upper back	-0.25 (1.55)	0.249	-0.11 (1.39)	0.634	0.476
Lower back	+0.06 (2.08)	0.837	+0.15 (1.99)	0.664	0.552

## Discussion

The main result of this workplace randomized controlled trial consisting of diet, physical exercise and cognitive behavioral training was a mean weight loss of 3.6 kg in the intervention group. In addition, a substantial effect was found for systolic and diastolic blood pressure with decreases of 7.5 and 5.4 mmHg, respectively. A remarkably large adherence was obtained with only seven out of 98 participants dropping out during the three-month intervention. The results will be discussed in more detail below.

Among the 105 female health care workers, 93% was overweight, showing that efficient weight loss programs are highly relevant as health promotion for this sector. To our knowledge, this is the first randomized controlled workplace intervention among health care workers with the specific aim to reduce body weight. One previous Danish workplace health promotion study among health care workers, consisting of 20 weeks with weight training, fitness training and advice on healthy living did not show any positive effects on body weight [40]. In a non-randomized study by Rigsby and colleagues from 2009 among 454 female employees at a hospital and nursing home, eight weeks weight loss intervention in groups reduced mean body weight by 3.8 kg [41]. Other workplace studies not specifically targeting health care workers but aiming at weight loss with intervention periods from 10 to 16 weeks have shown weight losses from 1.3 - 4.5 kg [42-45]. In comparison to these workplace studies targeting similar populations or using comparable intervention programs, the present study shows an equal or even larger effect. Also the decrease in blood pressure was in line with or even larger than reported in previous studies on weight loss and blood pressure [46].

The intervention consisting of diet, physical exercise and cognitive behavioral training during working hours one hour/week was shown to be very effective, generating a significant weight loss, decreased blood pressure and increased aerobic fitness after three months. These findings support the recommendations of combining these three initiatives for successful weight loss [23]. However, the long-term effects of this combined intervention remain to be investigated.

The intervention was not able to increase muscle strength, indicating that no changes in muscle mass occurred. Increasing muscle mass was considered a means to further ease weight loss by raising resting metabolism. Therefore, 10 - 15 minutes per week of physical exercise seems insufficient if the aim is to increase muscle strength while simultaneously encouraging weight loss. However, it was sufficient to maintain muscle strength alongside the weight loss. Similarly, no increase in VO_2 _max was found. However due to the weight loss, the aerobic fitness being relative to the body weight was increased. The maintained physical capacity in combination with the reduction in body fat % indicates that the weight loss achieved during the intervention is primarily due to loss of fat tissue. The increased aerobic fitness may represent a functional benefit, decreasing the relative physical workload of the health care workers, and therefore their risk of cardiovascular disease [47].

Only seven participants dropped out during the three months intervention. The adherence rate was therefore higher than in most other weight loss studies at the workplace [48,49]. The successful adherence may be due to a number of initial precautions taken. First, workplaces adopting this intervention study were obliged by contract to provide time for the intervention during working hours. Second, each of the seven intervention groups was, as far as possible, guided by a single instructor to personalise the interventions. Third, a close collaboration between managers ensured that obstacles for the intervention were quickly solved. In summary, the workplace approach is likely to explain the high adherence and therefore the positive results of the study. Other studies have pointed out that workplace-initiated weight loss programs promote a team spirit among the employees [50,51]. The participants tend to form into particular groups at workplaces, often based on gender, educational backgrounds and interests, which makes group counseling easier. The participants see each other on a daily basis during the intervention period and tend to share meals and have opportunities to meet immediately after work for exercise [41]. In the present study, employees without weight problems were also invited to take part in the intervention. Not excluding them from the intervention may have contributed to a positive team spirit regarding the initiative.

The present study was conducted as a cluster randomized single-blinded controlled trial. It was carried out at a workplace that enabled us to target a high-risk group and obtain a very high adherence. The results in this paper were tested using intention-to-treat analyses (ITT), where missing observations are carried forwards or backwards. In spite of this conservative approach, we were able to reveal significant effects on weight loss and related outcomes such as fat percentage, waist circumference and blood pressure.

For a weight reduction program, a three months perspective is a short time frame. This study showed strong results after three months, but the main aim of the project is to maintain the weight loss for a longer period of time. Maintenance of the improved bodyweight, blood pressure and aerobic fitness is well known to reduce the risk of chronic diseases such as cardiovascular diseases and Type 2 diabetes, which in turn may reduce the risk for sick leave [52]. There was no observed effect on musculoskeletal pain after three months of the intervention. However, because weight loss will lower the mechanical load on joints and potentially improve work postures, it may have a positive effect on musculoskeletal pain in the long run.

A limitation in the study is the lack of quantitative registration of physical training doses in leisure time. The logbook was primarily used to facilitate the individual coaching and serve as a motivating factor. Another limitation is that in the integrated multiple intervention concept of this study the importance of each of the components cannot be evaluated. A four-armed design where each of the components as well as the combined concept is tested against a control group would have been ideal, but also unrealistic with the current resources and the workplaces available. A qualitative process analysis with focus group interview is another approach that would have been possible, but unfortunately not performed. Finally, the target group only consists of females and the results cannot be extrapolated to males. Concerning statistics, several ANCOVA models were carried out for testing effects of the intervention on multiple outcomes. The risk for a chance finding may therefore be resent. However, reducing the level of significance would substantially increase the risk for a type II error. This aspect ought to be included in the interpretation of the study results.

## Conclusions

This workplace-initiated intervention enabled us to target a high-risk group. The combination of diet, physical exercise and cognitive behavioral training resulted in significant weight loss, decreased blood pressure and increased aerobic fitness after three months. The positive results are encouraging regarding the use of workplace initiated weight loss interventions. The long-term effects of the intervention remain to be investigated.

## Competing interests

The authors declare that they have no competing interests.

## Authors' contributions

JRC, KS, KO and AHO designed and concepted the study. JRC designed the diet and the physical exercise protocol. JRC and AF together designed the cognitive behavioral training protocol. JRC was responsible for the work place, participant recruitment, intervention, test sessions, data collection and statistical analyses and together with DEK performed the data processing. All authors were involved in data interpretation. JRC wrote the first draft, and all authors read and approved the final manuscript.

## Pre-publication history

The pre-publication history for this paper can be accessed here:

http://www.biomedcentral.com/1471-2458/11/671/prepub
